# Immune Reconstitution in Pediatric Patients Following Hematopoietic Cell Transplant for Non-malignant Disorders

**DOI:** 10.3389/fimmu.2020.01988

**Published:** 2020-08-18

**Authors:** Sima T. Bhatt, Jeffrey J. Bednarski

**Affiliations:** Department of Pediatrics, Washington University School of Medicine in St. Louis, St. Louis, MO, United States

**Keywords:** immune reconstitution, hematopoietic stem cell transplant, non-malignant disorders, hemoglobinopathy, severe combined immunodeficiency, aplastic anemia

## Abstract

Allogeneic hematopoietic cell transplant (HCT) is curative for pediatric patients with non-malignant hematopoietic disorders, including hemoglobinopathies, bone marrow failure syndromes, and primary immunodeficiencies. Early establishment of donor-derived innate and adaptive immunity following HCT is associated with improved overall survival, lower risk of infections and decreased incidence of graft failure. Immune reconstitution (IR) is impacted by numerous clinical variables including primary disease, donor characteristics, conditioning regimen, and graft versus host disease (GVHD). Recent advancements in HCT have been directed at reducing toxicity of conditioning therapy, expanding donor availability through use of alternative donor sources, and addressing morbidity from GVHD with novel graft manipulation. These novel transplant approaches impact the kinetics of immune recovery, which influence post-transplant outcomes. Here we review immune reconstitution in pediatric patients undergoing HCT for non-malignant disorders. We explore the transplant-associated factors that influence immunologic recovery and the disease-specific associations between IR and transplant outcomes.

## Introduction

Allogeneic hematopoietic cell transplant (HCT) is a key therapeutic approach for many non-malignant hematopoietic diseases in pediatric patients, including hemoglobinopathies, bone marrow failure syndromes, and immunodeficiencies. Effective reconstitution of donor-derived innate and adaptive immune cell number and function following HCT is critical for promoting donor cell engraftment, restoring protection against infections, and improving overall survival (1, 2).

Recovery of immunity after HCT is influenced by various clinical factors, including primary diagnosis, donor type, stem cell source, graft manipulation, conditioning regimen (i.e., intensity of conditioning, use of irradiation, serotherapy), and pharmacologic prophylaxis, development and treatment of graft-versus-host disease (GVHD) (1, 2). After HCT, establishment of donor immunity is variable and occurs in phases. Innate immune reconstitution (IR) occurs first with neutrophils, monocytes, natural killer (NK) cells, and dendritic cells expected to normalize in the first weeks to month after HCT (1). Adaptive immune system recovery occurs more slowly with B cell and CD8 T cell numbers normalizing between 100 days and 6 months post HCT and thymic-dependent CD4 T cell reconstitution occurring between 6 and 9 months (1). Initial T cell reconstitution occurs through peripheral expansion of CD8 memory T cells from the donor graft or recipient T cells remaining after conditioning (3). These peripherally expanded CD8 T cells are responsive to cytokines and previously encountered viruses; however, they have limited ability to respond to novel antigens (3). The second phase, leading to full T cell reconstitution, relies on lymphoid progenitors undergoing thymic differentiation into naive CD4 or CD8 T cells expressing MHC-restricted, antigen-specific T cell receptors (3). The kinetics of reconstitution of these distinct components of the immune system correlate with post-transplant morbidity related to infections, graft loss and GVHD. Here we review the factors that influence recovery of innate and adaptive immunity in pediatric patients undergoing HCT for non-malignant disorders and the impact of this reconstitution on general and disease-specific outcomes.

## Transplant-Associated Factors Affecting Immune Reconstitution

### Stem Cell Source

Peripheral blood (PB), bone marrow (BM), or umbilical cord blood (UCB) stem cells can be utilized for HCT from either related (RD) or unrelated donors (URD). These donor sources vary in cellular composition with PB grafts having 10-fold higher T and B cells than BM grafts and single UCB grafts having 10–100-fold fewer nucleated cells compared to BM (1, 4, 5). The differences in graft composition impact donor IR and infectious complications following HCT. Regarding innate immunity, neutrophil engraftment occurs at approximately 14, 21, and 30 days after a PB, BM, and UCB HCT, respectively (6). Interestingly, NK cell numbers normalize by 1 month post HCT independent of graft source (6). Yet, UCB recipients have been found to have higher numbers of NK cells at 3, 9, and 12 months after transplant (7).

Graft source also impacts reconstitution of adaptive immunity. HCT with UCB has been associated with higher naive and memory B cell numbers at 6 months post HCT compared to BM and PB grafts (8). In contrast, T cell reconstitution is delayed after UCB HCT (7–9). UCB contains antigen-inexperienced naive T cells; therefore, T cell recovery is entirely thymic dependent resulting in profound early lymphopenia (7, 10, 11). Recipients of UCB HCTs have a slower recovery of thymopoiesis than patients receiving BM stem cells as evidenced by a lower thymic-derived naive CD4 T cells at 6 months post HCT (7).

**FIGURE 1:**
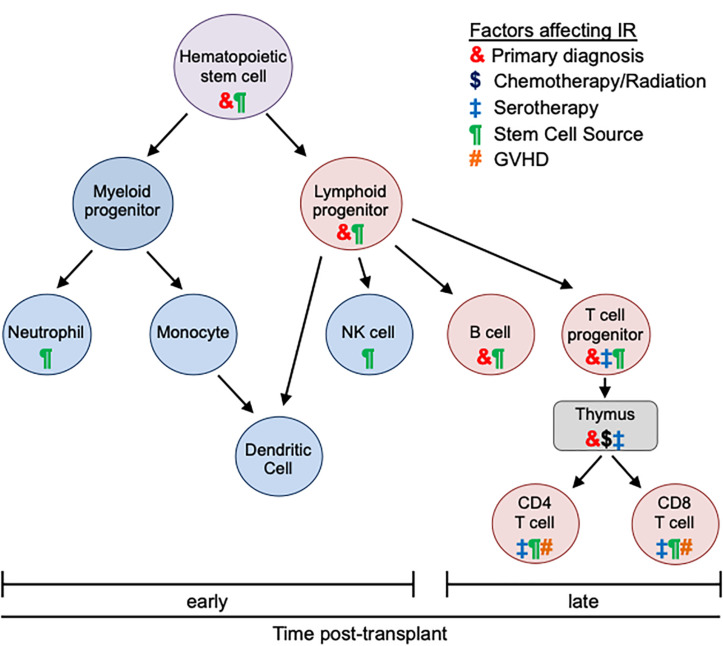
Effects of transplant-related factors on immune reconstitution. Different types of immune cells and their differentiation are depicted. After allogeneic HCT innate immunity (blue) recovers early (within 30 days). Reconstitution of adaptive immunity (red) is later and more variable (often up to 1 year). The kinetics of immune recovery is influenced by primary diagnosis (&), conditioning regimen ($), use of serotherapy (‡), stem cell source (¶), and GVHD (#). Each transplant-associated factor distinctly impacts different immune populations and differentiation stages.

T cell reconstitution also differs between BM and PB recipients. In a single institution randomized trial, patients who received PB grafts had faster lymphocyte recovery, most significantly CD4 T cells, compared to BM graft recipients (4). Consistent with slower IR, BM stem cell recipients had a 2.4-fold higher rate of severe infections and a higher risk of infection-related mortality (4). A larger, phase 3 trial confirmed earlier IR and lower infection risk in patients receiving PB grafts but did not identify any differences in mortality (12). Thus, donor IR after HCT is highly impacted by distinct properties of the different stem cell sources (Figure 1).

### Alternative Donor Sources

While an HLA matched donor is preferred, less than 25% of patients will have an available sibling donor and the likelihood of identifying a matched URD in the registry is impacted by numerous factors, including ethnicity of the patient (13). Consequently, alternative donors have been increasingly used for HCT with unique implications for post-HCT IR (Figure 1).

UCB has been utilized as an alternative donor source and has distinctive IR properties as discussed above. However, there are significant barriers to success of UCB transplants, including graft failure and delayed neutrophil and T cell recovery, resulting in infectious complications (10, 11, 14). Addressing these obstacles has been an active area of investigation (6, 10, 15). UCB has lower total nucleated cell and CD34 + cell dose (per recipient’s weight), which has been associated with delayed hematological recovery and graft failure (11). Strategies to improve cell dose for UCB have included double cord blood transplant and *ex vivo* expansion of cord blood units. While IR data on double UCB HCT is limited in pediatrics, in adults, it has not consistently demonstrated an improvement in IR compared to single UCB HCT (10, 16, 17). This may be, in part, related to confounding factors, including the use of T cell depletion (10). Further studies are needed to better address this question. In contrast, recent early phase clinical trials using *ex vivo* cord blood expansion have demonstrated that neutrophil engraftment can be shorted to 9 days from 21 days (15, 18). In regard to T cell recovery, lower doses of anti-thymocyte globulin (ATG) have been associated with faster recovery of CD4 and CD8 T cells after UCB transplant (14, 19, 20). Additionally, use of better HLA-matched cord blood units with higher CD3 T cell counts has been shown to improve immune recovery (21).

The use of haploidentical donors as an acceptable alternative stem cell source has surged with recent studies aimed to reduce the risk of GVHD, sustain donor engraftment, and support earlier IR (13, 22). The kinetics of IR following haploidentical donor HCT depends on conditioning regimen, stem cell source, and graft manipulation strategy utilized. For example, time to neutrophil engraftment varies from a median of 11–12 days after T cell depletion with high dose CD34 + cells to 13 days after GCSF-mobilized haploidentical unmanipulated PB graft to 15 days after unmanipulated haploidentical BM (23). Similar to HLA-matched transplant, monocyte and NK cell recovery is rapid and occurs by day 15 and 30, respectively, after haploidentical HCT (23). Regarding adaptive immunity, patients receiving T cell replete haploidentical grafts have more rapid T cell IR during the first 6 months after HCT compared to patients who received T cell-depleted grafts (23). T cell function and new naive T cell production remain low for 12–24 months after unmanipulated haploidentical HCT (23).

Due to delayed recovery of adaptive immunity and associated infection risks, strategies for *ex vivo* elimination of αβ T cells and CD19 B cells with no pharmacologic prophylaxis for GVHD has been utilized for haploidentical transplant in patients with non-malignant disorders (13, 22). In a study of 23 patients, γδ T cell recovery occurred early (∼1 month post HCT), but αβ T cell and CD19 B cell repopulation was delayed to 9–12 months, respectively (13). Alternative donor sources are often used in patients with non-malignant disorders who have no available familial or registry donor. Improving IR in this patient population remains an active area of investigation.

### Conditioning Strategies

IR is also impacted by conditioning regimen, including intensity of chemotherapy, use of radiation, and use of serotherapy (Figure 1). In particular, conditioning therapy can damage the thymus and impair its function, which is essential for full T cell reconstitution. For example, cyclophosphamide and radiation induce acute thymic injury with loss of cellularity whereas ATG and alemtuzumab serotherapy significantly deplete thymocytes resulting in prolonged T cell aplasia (3).

Patients with non-malignant disorders often receive reduced toxicity (RTC) and reduced intensity conditioning (RIC) regimens in order to limit the morbidity associated with myeloablative conditioning (MAC). RIC regimens are non-myeloablative while RTC regimens are myeloablative. Both approaches have fewer side effects and organ toxicities compared to traditional MAC. Law et al. reported that following a RTC regimen of alemtuzumab, busulfan, and fludarabine median time to neutrophil recovery was 16 days while time to B cell and T cell reconstitution was 3 and 6 months, respectively (24). A RIC approach with alemtuzumab, fludarabine and melphalan has been used by our group and others (25–28). We recently reported IR and infectious complications in patients after HCT with early alemtuzumab (day -21) (26). NK cell recovery was rapid by day 100 and lymphocyte recovery was dependent on donor source, namely related (RD) versus unrelated donor (URD). Mean CD3, CD4, and CD8 T cell numbers normalized by 6 months after RD HCT and by 1 year in the URD group (26). B cell recovery occurred by day 100 for RD recipients and by 1 year for URD recipients (26). Despite these differences, infections did not differ between the groups (26).

Timing and dose of serotherapy significantly impact IR (20, 29, 30). Admiraal et al. reported on IR in patients with malignant and non-malignant disorders receiving ATG as part of conditioning (20). They found that successful CD4 IR was related to the area under the curve (AUC) of ATG after donor stem cell infusion (20). Patients who received UCB HCT had delayed IR with an AUC ≥ 20 AU × day/mL while patients who received BM and PB HCT had decreased IR only at an AUC ≥ 100 AU × day/mL (20). Notably, an ATG AUC ≥ 40 AU × day/mL prior to stem cell infusion resulted in a lower incidence of graft failure and acute and chronic GVHD (20). Marsh et al. similarly demonstrated that alemtuzumab level at time of transplant impacts outcomes (30). They found patients with a level <0.15 mg/mL had threefold higher rates of acute GVHD than patients who had levels >0.16 mg/mL at the time of transplant. Alemtuzumab levels above 0.57 mg/mL were associated with delayed T cell recovery and very high levels (4 mg/mL) were associated with mixed chimerism (30). The approach to conditioning is often dictated by primary disease/graft source and requires careful consideration to balance IR with risks of GVHD and graft failure.

## Disease-Specific Outcomes

### Hemoglobinopathies

HCT for pediatric patients with thalassemia and sickle cell disease is potentially curative and the impact of IR on transplant-associated morbidity and outcome has been investigated by several groups. Rajasekar et al. detailed IR patterns in patients with β thalassemia major following MAC and matched related donor (MRD) HCT with BM graft (31). They found that NK cells, monocytes and dendritic cells recovered within 1 month of transplant (31). CD8 T cells and B cells repopulated at 2 and 4 months, respectively, while CD4 T cell recovery did not occur by 1 year post HCT (31). Consistent with this, naive CD4 T cell (CD45RA^+^) recovery was delayed more than a year and correlated with age, with younger patients having faster recovery (31). Interestingly, multivariate analysis showed that NK cell count correlated with transplant success as patients with NK cells below a median of 142/μL at 28 days post HCT had a significantly higher rejection rate and lower event free survival (31).

In order to prevent graft failure/rejection, *in vivo* T cell depletion is increasingly utilized in patients with hemoglobinopathies (32). An evaluation of IR in children with severe β thalassemia major following matched sibling donor (MSD) HCT found that the addition of ATG led to delayed CD8 T cell recovery at 6 months but no change in CD4 T cell reconstitution, which occurred at 12 months (33). Use of ATG containing conditioning regimens was associated with variable rates of bacterial infection (17–70%) and cytomegalovirus (CMV) reactivation (36–45%) (32, 33). These infectious complications are similar to those in patients transplanted without *in vivo* T cell depletion (32). However, rates of GVHD were lower after ATG-based conditioning (32).

Our group has reported similar outcomes in patients with hemoglobinopathies undergoing HCT with *in vivo* T cell depletion utilizing alemtuzumab (34). Lymphocyte recovery of CD4, CD8, and CD19 occurred by 1 year post transplant and was impacted by duration and intensity of immunosuppression for GVHD prophylaxis/treatment (34). Infection risk was highest in the first 6 months post HCT with bacterial infections and CMV reactivation in 28 and 43% of patients, respectively (34).

### Aplastic Anemia

Patients with severe aplastic anemia undergo HCT as first line therapy if a MSD is available or as salvage therapy if they fail immune suppression therapy. A retrospective review of patients who failed immune suppression therapy and received MUD HCT after fludarabine, cyclophosphamide, and alemtuzumab conditioning therapy demonstrated that the majority of children achieved normal lymphocyte subsets by 12 months post HCT (35). Infectious complications included adenoviremia (2.3%), EBV viremia (22.7%), and CMV viremia (22.7%) (35). Our group published a report of 17 patients undergoing HCT with alemtuzumab, fludarabine and melphalan conditioning (36). While NK cells recovered early, T cell (both CD4 and CD8) and B cell recovery was markedly delayed with all populations normalizing by 1 year after HCT (36). Consistent with these kinetics, infection rates were higher in the first 6 months post HCT (36).

A recent study of pediatric and adult patients (median age of 14 years) with aplastic anemia treated with haploidentical HCT utilizing busulfan, cyclophosphamide and ATG reported rapid neutrophil recovery at median of 12 days and monocyte recovery by 30 days after transplantation (37). CD8 T cell recovery occurred at 60 days while CD4 T cell repopulation was delayed to 1 year post HCT, resulting in an inverted CD4:CD8 ratio during that time period (37). Interestingly, patients with a lower CD4:CD8 ratio on day 30 post HCT had higher overall survival (37). Younger recipient age, female gender, high mononuclear cell count in the graft, and absence of CMV reactivation were all independently associated with improved IR after transplant (37).

### Primary Immunodeficiency

Severe combined immunodeficiencies (SCID) are a heterogeneous group of genetic disorders characterized by a lack of T cell progenitors available to develop within the thymus resulting in failure of T cell maturation as well as impaired cellular and humoral immunity (38). IR following HCT for SCID is variable based on intrinsic factors related to the underlying genetic defect (i.e., timing of developmental arrest) and modifiable factors, such as conditioning therapy (Table 1) (38–41). HCT without conditioning from an HLA-matched donor (related or unrelated) or T cell-depleted haploidentical donor allows successful thymopoiesis and T cell IR in SCID patients with IL2 receptor gamma chain (*IL2RG*), Janus-associated kinase 3 (*JAK3*), and adenosine deaminase (*ADA*) mutations (38). However, patients with *IL2RG-* and *JAK3-*mutant SCID transplanted without conditioning have lower (often absent) donor stem cell engraftment and, consequently, do not have donor B cell repopulation (42). In the absence of donor B cell engraftment, patients often require lifelong immunoglobulin replacement. In contrast, patients with interleukin-7 receptor (*IL7R*)-deficient SCID have intact function of B cells, which can produce immunoglobulin with help from donor T cells (38). Notably, without donor stem cell engraftment, patients are at risk of early T cell exhaustion due to limited donor-derived thymopoiesis (38). In *ADA*-deficient SCID, the majority of patients who receive non-conditioned MRD HCT graft engraft donor stem cells and have sustained cellular and humoral IR (43). SCID patients with mutation of *RAG1*, *RAG2* or *DCLRE1C* (*ARTEMIS*) have arrest of thymopoiesis at later developmental stages and require conditioning to achieve recovery of donor immunity (38).

**TABLE 1:** Immune reconstitution with and without conditioning for SCID.

Genotype	Immune phenotype	Conditioning	CD8 T Cell	CD4 T Cell	B Cell	References
IL2RG/JAK3	T- B + NK-	No	+	+	–	(38–42, 44, 45)
		Yes	+	+	+	

ADA	T- B- NK-	No	+	+	+	
		Yes	+	+	+	

RAG1/2/Artemis	T- B- NK +	No	–	–	–	
		Yes	+	+	+	

IL7R	T- B + NK +	No	+	+	+*	
		Yes	+	+	+	

+Indicates reconstitution is likely after HCT. −Indicates unlikely to reconstitute after HCT. *Indicates recipient reconstitution aided by donor cells.

A recent prospective study demonstrated that patients with SCID who received conditioning (RIC or MAC) prior to HCT had significantly higher levels of T, B, and myeloid cell donor chimerism at day 100, which persisted at 1 year post HCT (44). Furthermore, use of conditioning correlated with higher CD4 cell counts and greater likelihood of independence from immunoglobulin therapy at 1 year post HCT (44). There was no difference in overall survival based on receiving conditioning (44). While IR is improved with pre-transplant conditioning, there are significant potential toxicities and optimal conditioning therapy is still not known (38, 44).

In addition to conditioning, many other variables impact IR after HCT in SCID patients. HCT with an URD is associated with better T cell reconstitution whereas HCT with a mismatched related donor has poorer B cell reconstitution (45). IR also varies based on SCID genotype. *RAG1/2* and *DCLRE1C* mutations have poorer T cell reconstitution after transplant (45). In regard to B cell reconstitution, in non-MSD recipients, *ADA*, *IL7R*, *CD45*, and *CD3* genotypes have a higher probability of stopping immunoglobulin replacement therapy compared to *IL2RG*, *JAK3*, *RAG1/2*, and *DCLRE1C* genotypes (45).

Regardless of genotype or conditioning, a CD4 T cell count ≥500 cells/cumm at 6 and 12 months post HCT correlates with significantly better long-term overall survival (45). Furthermore, in SCID patients receiving T cell replete grafts, low numbers of total T cells, CD8 T cells, naive CD4 T cells, and polyclonal Vβ diversity at day 100 were all linked to higher risk of death or need for a second transplant at 2 years (44).

## Discussion

Reconstitution of the donor-derived immune system is essential for achieving optimal outcomes for pediatric transplant recipients. The timing and extent of recovery of immune cell numbers and function directly impact infectious complications, development and treatment of GVHD, and long-term survival. Innate immunity establishes rapidly after transplant and, generally, is only modestly impacted by transplant-associated variables. In contrast, adaptive immunity recovers with highly variable kinetics that are strongly influenced by numerous factors. Indeed, the timing and characteristics of IR can be adjusted by modifiable factors, including stem cell source and dose, conditioning regimen, and use/timing of serotherapy. The establishment of donor immunity uniquely impacts the post-transplant course based on initial diagnosis and disease presentation. As such, it’s critical to not only assess general patterns of IR but to evaluate these within disease-specific contexts.

Newer transplant approaches utilizing alternative donor sources, novel preparative regimens, and innovative graft manipulation strategies will invariably impact recovery of immune function. Additionally, identifying therapies that enhance IR remains an important focus of investigation. Innovative approaches include use of cytokines (IL-7 and IL-22), keratinocyte-growth factor, sex steroid ablation, and adoptive cell therapies (3, 46–51). Cellular therapies, such as viral-specific T cells, provide opportunities to support immune function while awaiting establishment of full IR. Careful evaluation of immune recovery will be essential in determining the impact of these therapeutic advances on transplant outcomes.

## Author Contributions

SB and JB wrote the manuscript jointly. Both authors contributed to the article and approved the submitted version.

## Conflict of Interest

The authors declare that the research was conducted in the absence of any commercial or financial relationships that could be construed as a potential conflict of interest.

## Abbreviations

ATG: anti-thymocyte globulin
BM: bone marrow
CMV: cytomegalovirus
GVHD: graft-versus-host disease
HCT: hematopoietic cell transplant
IR: immune reconstitution
MAC: myeloablative conditioning
MRD: matched related donor
MSD: matched sibling donor
NK: natural killer
PB: peripheral blood
RD: related donor
RIC: reduced intensity conditioning
RTC: reduced toxicity conditioning
UCB: umbilical cord blood
URD: unrelated donor.
